# Epstein-Barr Virus Evades CD4^+^ T Cell Responses in Lytic Cycle through BZLF1-mediated Downregulation of CD74 and the Cooperation of vBcl-2

**DOI:** 10.1371/journal.ppat.1002455

**Published:** 2011-12-22

**Authors:** Jianmin Zuo, Wendy A. Thomas, Tracey A. Haigh, Leah Fitzsimmons, Heather M. Long, Andrew D. Hislop, Graham S. Taylor, Martin Rowe

**Affiliations:** Cancer Research UK Birmingham Cancer Centre, University of Birmingham, Birmingham, United Kingdom; Emory University, United States of America

## Abstract

Evasion of immune T cell responses is crucial for viruses to establish persistence in the infected host. Immune evasion mechanisms of Epstein-Barr virus (EBV) in the context of MHC-I antigen presentation have been well studied. In contrast, viral interference with MHC-II antigen presentation is less well understood, not only for EBV but also for other persistent viruses. Here we show that the EBV encoded BZLF1 can interfere with recognition by immune CD4^+^ effector T cells. This impaired T cell recognition occurred in the absence of a reduction in the expression of surface MHC-II, but correlated with a marked downregulation of surface CD74 on the target cells. Furthermore, impaired CD4^+^ T cell recognition was also observed with target cells where CD74 expression was downregulated by shRNA-mediated inhibition. BZLF1 downregulated surface CD74 via a post-transcriptional mechanism distinct from its previously reported effect on the CIITA promoter. In addition to being a chaperone for MHC-II αβ dimers, CD74 also functions as a surface receptor for macrophage Migration Inhibitory Factor and enhances cell survival through transcriptional upregulation of Bcl-2 family members. The immune-evasion function of BZLF1 therefore comes at a cost of induced toxicity. However, during EBV lytic cycle induced by BZLF1 expression, this toxicity can be overcome by expression of the vBcl-2, BHRF1, at an early stage of lytic infection. We conclude that by inhibiting apoptosis, the vBcl-2 not only maintains cell viability to allow sufficient time for synthesis and accumulation of infectious virus progeny, but also enables BZLF1 to effect its immune evasion function.

## Author Summary

Epstein-Barr virus (EBV) is a herpesvirus and an important human pathogen that can cause diseases ranging from non-malignant proliferative disease to fully malignant cancers of lymphocytes and epithelial cells. The persistence of EBV in healthy individuals relies on the balance between host immune responses and viral immune evasion. As CD4^+^ immune T cell responses include both helper and cytotoxic functions, viral mechanisms for interfering with MHC class II antigen presentation to CD4^+^ T cells have the potential to greatly influence the outcome of viral infections. Our work on Epstein-Barr virus provides a new paradigm for viral immune evasion of MHC-II presented antigen by targeting CD74. CD74 is a dual function protein; it serves as a surviving receptor as well as a chaperone for MHC-II antigen presentation. Therefore, downregulation of CD74 as a T cell evasion strategy comes at the cost of potentially inducing cell death. However, EBV also encodes a vBcl-2 to attenuate the toxicity associated with reduced CD74, thus enabling the immune-impairment function to be effected. We expect that future studies will identify other viruses utilizing a similar strategy to evade CD4^+^ immune T cell responses.

## Introduction

Successful persistence of viral infection depends on the establishment of a balance between host immune responses and viral immune evasion. Epstein-Barr virus (EBV), which is carried by more than 90% of the adult human population worldwide, is a prime example of a persistent virus that is generally harmless, but which can cause serious disease including various tumours [1].

A number of immunoevasins of EBV have recently been identified as acting at different points along the MHC class I (MHC-I) presentation pathway to modulate recognition by CD8^+^ T cell responses [2]–[7]. However, with regards to evasion of the MHC class II (MHC-II) antigen presentation pathway, EBV is less well understood. Virus-specific CD4^+^ T cell responses, which include some clones with cytotoxic activity, are broadly distributed against numerous proteins encoded by the EBV genome; both latent protein antigens [8] and the larger number of lytic protein antigens [9], [10]. These observations indicate a need for EBV to also modulate MHC-II antigen presentation pathways, particularly during in EBV lytic cycle.

Mechanisms for interfering with the MHC-II antigen presentation pathway have been reported for other herpesvirus; for example, US2, US3 and pp65 of cytomegalovirus [11]–[13] and glycoprotein B (gB) of herpesimplex virus type 1 [14]. However, EBV has no homologues to these immune evasion genes of CMV, and there is no evidence that the homolog of HSV-1 gB protein encoded by the *BALF4* gene of EBV targets the MHC-II pathway. An unrelated EBV glycoprotein, gp42, has however been shown to associate with MHC-II molecules and to inhibit antigen presentation to CD4^+^ T cells [15], [16].

More recently, the immediate-early EBV gene *BZLF1*, which encodes a transcription factor initiating EBV lytic cycle, was reported to be a potential modulator of MHC-II antigen presentation [17]. Ectopic expression of BZLF1 in Raji cells inhibited the expression of MHC-II molecules, apparently through repression of CIITA transcription. However, interpretation of these data is complicated by the fact that Raji is an EBV-carrying B cell line in which expression of BZLF1 can initiate virus lytic cycle and, therefore, the expression of other viral genes that may be responsible for modulating MHC-II expression. One such candidate is the early antigen, BGLF5, which has been shown to induce global mRNA degradation and thereby to reduce expression of various host proteins, including MHC-I and MHC-II [2], [3]. In addition, as the level of expression of MHC molecules does not necessarily reflect the degree of T cell recognition, it is important to assay antigen presentation using functional T cell recognition.

In this study, we demonstrated for the first time that BZLF1 does indeed impair recognition by EBV-specific CD4^+^ T cells. However, this was not the result of downregulation of MHC-II α/β chain molecules as expected, but through downregulation of the invariant chain (Ii, or CD74) which serves as a chaperone to ensure correct loading of antigenic peptide fragments. As CD74 also mediates cell survival, its downregulation by BZLF1 induced cell death. A pivotal role was also identified for a vBcl-2 (BHRF1) during virus lytic cycle. By abrogating the concomitant toxic properties of BZLF1, the vBcl-2 enabled the immune evasion function of BZLF1.

## Results

### CD4^+^ T cell recognition is impaired in BZLF1 expressing LCLs

To gain a complete picture of effects on the antigen presentation pathway, it is crucial to include the functional T cell recognition assays as readout. We sought to determine whether BZLF1 can impair the MHC-II antigen presentation of newly processed MHC-II/peptide complexes and also of pre-existing MHC-II/peptide complexes. Recognition of newly processed MHC-II/peptide complexes was assayed by pulsing target cells with virus particles, before co-culturing with BXLF2-specific ‘LEK’ CD4^+^ effector T cells recognising a virion antigen. Pre-existing MHC-II/peptide complexes were assayed with EBNA1-specific ‘SNP’ CD4^+^ effector T cells to detect EBNA1 antigen that is expressed in both the latent and lytic phases of infection in EBV-transformed B lymphoblastoid cell lines (LCL). Whilst antigen processing for presentation via MHC-II complexes is generally considered to occur mainly through endocytosis of exogenous antigen (e.g. virus particles), it is also possible for endogenously expressed antigen to be processed by directly accessing the MHC-II antigen processing pathway [18], [19]. EBNA1 is paradigmatic for endogenous processing of target peptides for recognition by immune CD4^+^ T cells [18], [20].

**Figure 1:**
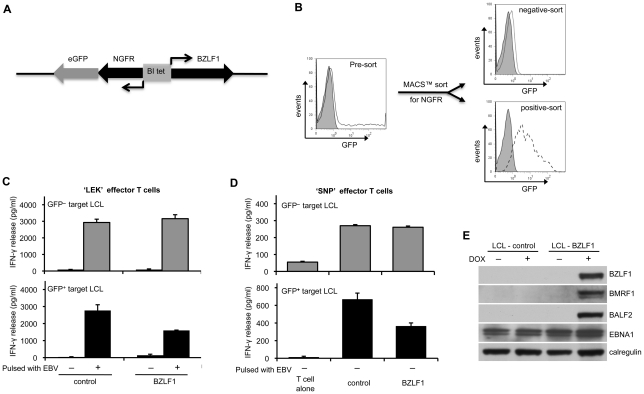
CD4^+^ T cell recognition is impaired in the BZLF1-expressing LCLs. (**A**) The expression plasmid pRTS-CD2-BZLF1 carries a bidirectional DOX regulatable promoter, BI-Tet, which drives the expression of BZLF1 together with a non-functional neuronal growth factor receptor (NGFR) and green fluorescent protein (GFP) as markers of DOX-induced expression. (**B**) BZLF1KO-LCLs transfected with pRTS-CD2-BZLF1 or pRTS-CD2-control vector were treated with DOX for 24 h. The induced plasmid-containing cells were separated using MACSelect LNGFR MicroBeads. Both the unlabeled cells and the labeled cells were collected, and then analyzed by flow cytometry. The flow-through cells were NGFR^–^/GFP^–^, indicating that they lacked the transfected plasmid and/or DOX treatment failed to induce expression from pRTS-CD2-BZLF1 (dotted line, top histogram). The bound and eluted cells were NGFR^+^/GFP^+^, which indicated that expression of BZLF1 from pRTS-CD2-BZLF1 had been induced (dashed line, bottom histogram). (**C**) These sorted cells were pulsed with or without concentrated EBV B95.8 virus particles and then were co-cultured with ‘LEK’ CD4^+^ effector T cells (specific for a BXLF2-derived peptide), for a further 18 h before assaying T cell recognition by IFN-γ ELISA. (**D**) Unpulsed targets were also assayed with ‘SNP’ CD4^+^ effector T cells (specific for a EBNA1-derived peptide). All results are expressed as IFN-γ release in pg/ml, and error bars indicate standard deviation of triplicate cultures. (**E**) The pRTS-CD2-control and pRTS-CD2-BZLF1 transfected LCLs were analyzed, before and after DOX induction, by SDS-PAGE and immuno-blotting with antibodies specific for BZLF1, BMRF1, BALF2, EBNA1 or calregulin as a loading control.

In our initial experiments we generated LCL targets with a BZLF1-knockout recombinant EBV (BZLF1KO-LCLs), and transfected them with a doxycycline (DOX)-inducible BZLF1-expressing vector, pRTS-CD2-BZLF1. This vector expresses a CD2 surface marker from the vector backbone, allowing enrichment of viable transfected cells at 24h after transfection. The BZLF1 gene and an NGFR-IRES-GFP sequence were regulated by a bi-directional DOX-regulated promoter, which allows a further round of purification of BZLF1-expressing cells following DOX-induction. Control BZLF1KO-LCL targets were transfected with a vector containing a non-sense reverse BZLF1 sequence insert. Details of the plasmid vectors and the isolation of transfected cells are described in Figure 1A. BZLF1- and control-vector transfected LCLs were treated with DOX for 24 h before separating the induced population according to NGFR expression. From each induced line, two populations were obtained: NGFR^–^/GFP^–^ cells lacking transfected plasmid, and NGFR^+^/GFP^+^ cells in which BZLF1/GFP (or control/GFP) expression was evident (Figure 1B). The different populations were used as target cells in CD4^+^ T cell assays where recognition was measured by assaying the release of IFN-γ from the effector T cells.

GFP^–^ cells from both the pRTS-CD2-control and the pRTS-CD2-BZLF1 transfectants were equally well recognised, both by BXLF2-specific CD4^+^ effectors following uptake and processing of virions (Figure 1C, upper histogram) and by EBNA1-specific CD4^+^ effectors (Figure 1D; upper histograms). In contrast, recognition of newly processed BXLF2 peptides from virus-pulsed targets (Figure 1C, lower histogram) and pre-existing peptides from EBNA1 (Figure 1D, lower histogram) was impaired in GFP^+^ cells expressing BZLF1 protein. The results illustrated in Figs. 1C and 1D are from one representative experiment. In three independent experiments, recognition of newly processed BXLF2 antigen by ‘LEK’ CD4^+^ T cells was reduced by 40–60%, and recognition of pre-existing EBNA1 peptide by ‘SNP’ CD4^+^ T cells was reduced by 25–40% following expression of BZLF1. These experiments demonstrate that BZLF1 expression in LCLs leads to impaired EBV-specific CD4^+^ effector T cell recognition.

In the BXLF2-specific CD4^+^ T cell recognition assays, there was very low but consistent recognition of BZLF1-expressing LCLs that had not been pulsed with EBV particles (bottom histogram, Figure 1C). This suggested that even within the short time-frame of this experiment the expression of BZLF1 can trigger lytic cycle and produce small but sufficient amounts of virus antigen for MHC-II processing. This conclusion was supported by further assays where expression of BZLF1 led to clear recognition not only by BZLF1-specific CD4^+^ effectors, but also by CD4^+^ effectors specific for the BMRF1 early antigen or the gp350 late antigen (Figure S1). Immunoblot analysis of the LCL targets showed that induction of BZLF1 led to expression of EBV lytic proteins (Figure 1E). Thus, whilst the data in Figure 1 are consistent with BZLF1 interfering with CD4^+^ T cell responses, further experiments with EBV-negative targets were necessary to avoid complications arising from BZLF1 initiation of the cascade of lytic cycle expression.

### Effect of BZLF1 on CD4^+^ T cell recognition of MHC-II presented antigen

To investigate the effect of BZLF1 on MHC-II antigen presentation in EBV-negative target cells, we developed a strategy that allowed analysis of transiently transfected target cells. As intercellular antigen transfer is never detectable for EBNA1 [20], we chose this as the target antigen. To increase the sensitivity of the assay, a mutant form of EBNA1 (Cyto-EBNA1) was used as its cytosolic location renders its processing to MHC-II molecules more efficient than wild-type nuclear EBNA1 [20].

**Figure 2:**
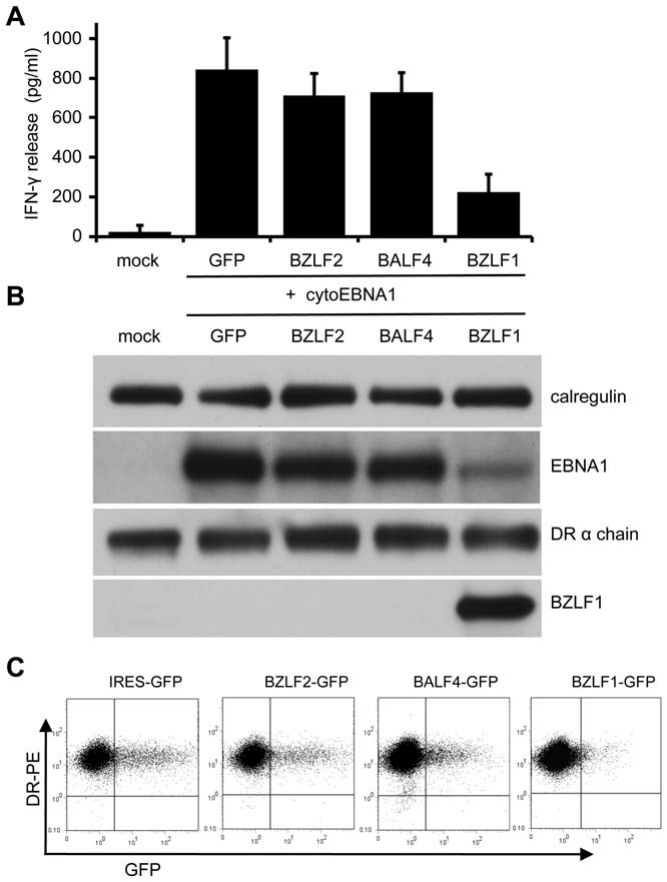
Effect of BZLF1 on CD4^+^ T cell recognition of MHC-II presented antigen. MJS-DRB5*01 cells were co-transfected with pCDNA-cytoEBNA1 expression vector together with IRES-GFP (GFP), BZLF2-GFP (BZLF2), BALF4-GFP (BALF4) or BZLF1-GFP (BZLF1) expression plasmids. (**A**) At 24 h post-transfection, the cells were used as targets for ‘SNP’ CD4^+^ effector T cells (specific for a EBNA1-derived peptide) as in Figure 1. Error bars indicate standard deviation of five independent experiments. (**B**) Total cell lysates of target cells were analyzed by immunoblotting using antibodies specific for EBNA1, DRα chain, BZLF1 or calregulin as a loading control. (**C**) Viable transfected target cells stained with PE-conjugated anti-DR, analyzed by flow cytometry.

EBV-negative MJS melanoma cells expressing MHC-II DRB5*01 were transfected with cyto-EBNA1 expression plasmid together with IRES-GFP plasmid vectors for BZLF1 or other EBV lytic genes. The cultures were then assayed for antigen presentation to MHC-II DRB5*01-restricted CD4^+^ T cells specific for the ‘SNP’ EBNA1-derived peptide. T cell recognition was measured by the release of IFN-γ from the effector cells. The results of 5 independent experiments are summarised in Figure 2A. Mock transfected cells were not recognised, but good recognition was seen with target cells co-transfected with cyto-EBNA1 and control IRES-GFP vector. Recognition of cyto-EBNA1 was not significantly affected by co-transfection of BZLF2 or BALF4 vectors expressing the EBV lytic proteins gp42 or gp110, respectively. However, recognition of cyto-EBNA1 was substantially and reproducibly reduced by 60–80% when the target cells were co-transfected with BZLF1.

Replicate aliquots of cells analyzed by immuno-blots confirmed expression of BZLF1 in the cells transfected with the BZLF1-GFP plasmid (Figure 2B). There was no obvious change of MHC-II DR expression in any of the target cells. Whilst the levels of EBNA1 were similar in the cells co-transfected with cyto-EBNA1 and IRES-GFP, BZLF2-GFP or BALF4-GFP plasmids, there was an unexpected and marked reduction of EBNA1 expression when co-transfected with the BZLF1-GFP plasmid (Figure 2B).

Two colour flow cytometry analysis of viable cells stained for surface MHC-II DR suggested that none of the EBV lytic genes tested reduced the level of cell surface DR molecules on GFP^+^ cells compared to the expression on untransfected GFP^–^ cells in the same cultures (Figure 2C). Notably, there were relatively few GFP^+^ cells in the cultures transfected with BZLF1-GFP; typically less than 1% compared with 15% in cultures transfected with control IRES-GFP plasmid. This indicated a toxic effect of BZLF1 in these cells, which could account for the reduced EBNA1 target antigen expression (Figure 2B) and also the reduced T cell recognition (Figure 2A).

### BZLF1 downregulates anti-apoptotic Bcl-2 family members

**Figure 3:**
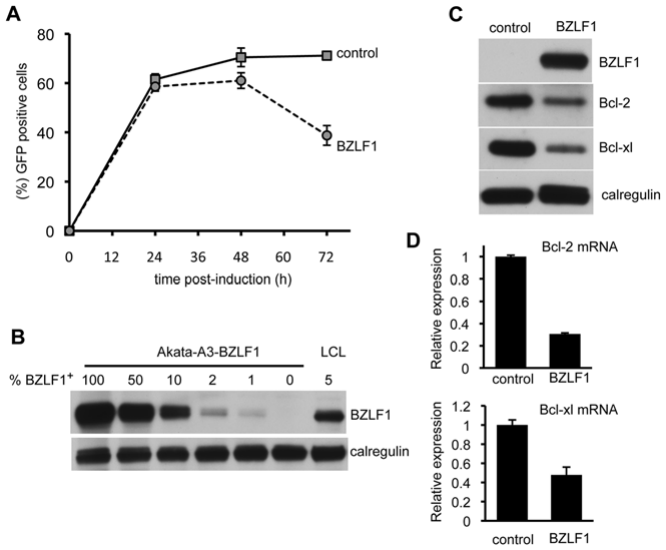
BZLF1 downregulates Bcl-2 family members. (**A**) EBV-negative Akata-A3 cells transfected with pRTS-CD2-control (solid line) or pRTS-CD2-BZLF1 (dotted line) were induced with DOX and analyzed by flow cytometry at the indicated times for the percentage of cells expressing GFP. Error bars indicate standard deviation of three independent induction experiments. (**B**) At 48 h after DOX induction, the GFP^+^ cells from control and BZLF1-transfected cultures were purified on a Mo-Flow cell sorter. The BZLF1 expressing Akata-A3 cell lysate was serially diluted with Akata-A3 cell lysate to give equivalent BZLF1 content of 100%, 50%, 10%, 2%, 1% or 0% and was analyzed by SDS-PAGE alongside a cell lysate from a spontaneous lytic LCL containing 5% BZLF1^+^ cells. The samples were immunoblotted with antibodies specific for BZLF1 or calregulin as a loading control. (**C**) Total cell lysates of the purified GFP^+^ populations from control- and BZLF1-transfected cultures were analyzed by SDS-PAGE and immunoblotting with antibodies specific for BZLF1, Bcl-2, Bcl-xl, or calregulin as a loading control. (**D**) Bcl-2 and Bcl-xl transcripts in total RNA isolated from induced and sorted cells were measured by QRT-PCR assay, which was normalized to measured GAPDH transcripts.

Toxicity of BZLF1 was confirmed in other cell types, including an EBV-negative subclone of the Akata Burkitt's lymphoma, Akata-A3. Here, we used the DOX-inducible pRTS-CD2-BZLF1 as in Figure 1. Akata-A3 cultures transfected with either pRTS-CD2-BZLF1 or pRTS-CD2-control plasmids were enriched by CD2 selection to around 80% purity. Following treatment with DOX, the percentage of GFP^+^ cells was monitored over a period of 72 h. For the first 24 h of DOX treatment, both control- and BZLF1-transfected cultures contained indistinguishable numbers of induced GFP^+^ cells, but thereafter the BZLF1 transfected cultures showed a drop in the percentage of GFP^+^ cells such that by 72 h these BZLF1 cultures contained only around half the percentage of GFP^+^ cells as the control cultures (Figure 3A). These results indicate that BZLF1 is toxic to Akata-A3, but with slower kinetics than in MJS cells. The slower kinetics of toxicity in Akata-A3 allowed examination of cellular protein expression at 48 h after BZLF1 induction. Pure populations of GFP^+^ cells were obtained by flow cytometer sorting for GFP expression, and were analysed by immunoblotting. The first important point to note is that physiological levels of BZLF1 were obtained in these cells. Titration of the A3-BZLF1 transfectants against an LCL containing 5% BZLF1^+^ cells (i.e. in lytic cycle), followed by densitometry analysis of immunoblots, indicated that the level of BZLF1 in the transfected cells was around 115% of that in lytically-infected LCLs (Figure 3B). Secondly blotting for Bcl-2 and Bcl-xL expression showed a clear downregulation of these anti-apoptotic proteins in the BZLF1-expressing Akata-A3 cells (Figure 3C), which correlated with a downregulation of Bcl-2 and Bcl-xl mRNA transcripts (Figure 3D).

### EBV vBcl-2 attenuates BZLF1 toxicity

**Figure 4:**
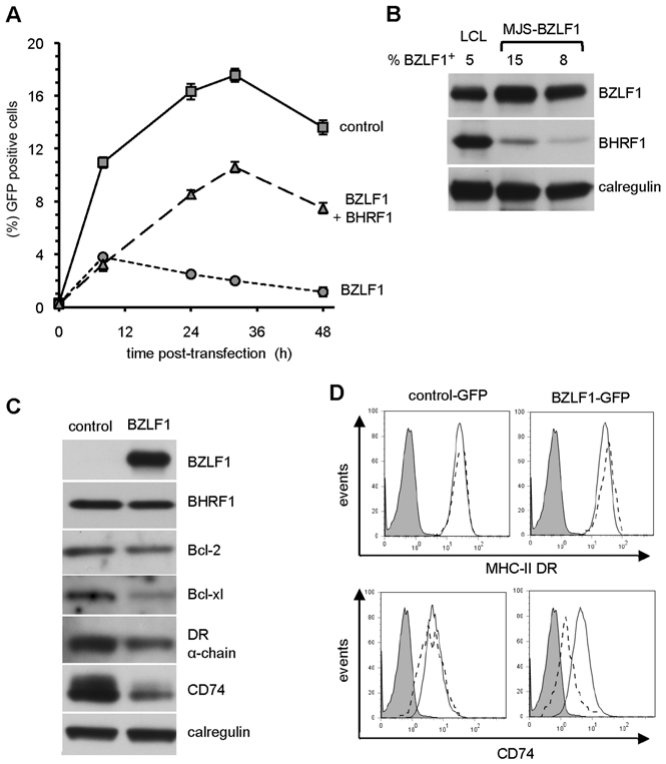
BHRF1 counteracts BZLF1 toxicity. (**A**) MJS cells transfected with control IRES-GFP (solid line), BZLF1-GFP (dotted line) or BZLF1-GFP together with BHRF1 plasmids (dashed line) were analyzed by flow cytometry at the indicated times for the percentage of cells expressing GFP. Error bars indicate the standard deviation of three independent transfections. (**B**) The cell lysates of transfected MJS cells from two independent transfections were analyzed by SDS-PAGE alongside lysates from a spontaneously lytic LCL. The samples were immuno-blotted with antibodies specific for BZLF1, BHRF1 or calregulin as a loading control. The percentage of BZLF1^+^ cells in each lysate is indicated above each lane of the blot. (**C**) MJS cells cotransfected with BHRF1 and either IRES-GFP (control) or BZLF1-GFP (BZLF1) expression plasmids were sorted for GFP on a Mo-Flow cell sorter at 48 h post-transfection. Total cell lysates of GFP^+^ transfected cells were analyzed by immuno-blotting using antibodies specific for BZLF1, Bcl-2, Bcl-xl, DRα chain, CD74, or calregulin as a loading control. (**D**) MJS cells cotransfected with BHRF1 and either IRES-GFP or BZLF1-GFP expression plasmids were stained with PE-conjugated anti-DR or with CD74 MAb followed by PE conjugated anti-mouse IgG2a antibody, then analyzed by flow cytometry. Histograms show the surface MHC-II DR or CD74 expression on GFP^–^ cells (solid line) and GFP^+^ cells (dashed line). The shaded histogram indicates isotype control staining.

EBV encodes a well-characterised Bcl-2 homolog, BHRF1, as an early lytic cycle protein [21]. We therefore investigated whether the toxicity of BZLF1 could be reversed by this vBcl-2. To address this possibility, we first examined the MJS line as it appears to be particularly sensitive to BZLF1. In the representative experiment shown in Figure 4A, MJS cells were transfected with IRES-GFP, BZLF1-GFP, or with BZLF1-GFP and a BHRF1 expression plasmid. Cultures were sampled at various time points post-transfection for analysis of GFP expression by flow cytometry. In the IRES-GFP control transfectants, about 10% of the viable cells were GFP^+^ at 8 h, rising to around 17% by 30 h. In marked contrast, MJS cells transfected with BZLF1-GFP showed only about 4% GFP^+^ viable cells at 8 h post-transfection, and thereafter the percentage of GFP^+^ cells gradually fell at later time points. However, when a BHRF1 expression vector was co-transfected with the BZLF1-GFP plasmid, the percentage of viable GFP^+^ cells continued to increase to about 10% by 30 h.

As in the previous set of experiments with Akata-A3 transfectants, the toxicity in MJS cells was mediated by physiological levels of BZLF1 (Figure 4B). Interestingly, the partial reversal of toxicity by co-expressed BHRF1 was achieved by levels of the vBcl-2 that were substantially lower than the levels observed in lytically-infected LCLs (Figure 4B). BHRF1-mediated protection from BZLF1-induced toxicity was also observed in other EBV-negative B cells (Figure S2). These results suggest that both the toxicity of BZLF1 and its reversal by BHRF1 are phenomena that are likely to be physiologically relevant during normal lytic cycle in EBV-infected cells.

### BZLF1 causes marked downregulation of CD74 but not MHC-II DR molecules at the cell surface

The protection afforded by BHRF1 in MJS cells enabled us to re-examine the effect of BZLF1 on MHC-II antigen presentation without the confounding effect of induced cell death. MJS cells co-transfected with BHRF1 together with either IRES-GFP or BZLF1-GFP were examined for expression of cellular proteins in viable GFP^+^ sorted subpopulations at 48 hr post-transfection. Immuno-blot analysis of cell lysates showed that, as with the Akata-A3 cells (Figure 3C), BZLF1 also downregulated Bcl-2 and Bcl-xl protein expression in MJS cells (Figure 4C).

Interestingly, whilst the total cellular level of MHC-II DRα molecules was only slightly reduced following BZLF1 expression, the level of invariant chain, CD74, was markedly downregulated (Figure 4C). In BZLF1-transfected cultures, cell surface MHC-II DR expression was reproducible slightly elevated by around 10–20% in the GFP^+^ gated cells (Figure 4D, top right histogram); in contrast, there was a marked reduction in the expression of CD74 (about 50%) on the surface of GFP^+^ cells compared with GFP^–^ cells (Figure 4D, lower right panel). One possible explanation for the discordance between total and cell surface MHC-II DR levels may be an altered localisation of MHC-II molecules when CD74 is downregulated. Analysis of control transfections showed that the levels of cell surface MHC-II DR (Fig 4D, top left histogram) and CD74 (bottom left histogram) were indistinguishable between GFP^+^ and GFP^–^ gated cells in the same culture.

**Figure 5:**
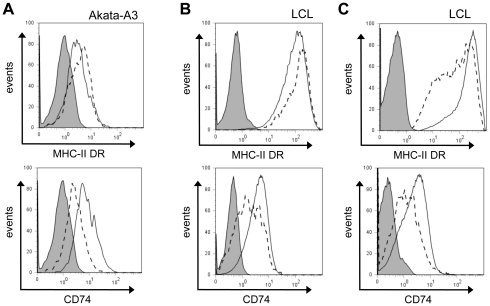
Downregulation of CD74, and not MHC-II DR, is a consistent effect of BZLF1. (**A**) EBV-loss Akata-A3 cells transfected with pRTS-CD2-BZLF1 or pRTS-CD2-control were induced with DOX for 24 h, then stained for MHC-II DR or CD74 as in Figure 4D. Histograms show the surface MHC-II DR and CD74 expression on GFP^+^ cells from pRTS-CD2-control transfection (solid line) and pRTS-CD2-BZLF1 transfection (dashed line). The shaded histogram is the isotype control staining. (**B**) An LCL with a subpopulation of spontaneously lytic cells was first stained for surface MHC-II DR or CD74 then fixed, permeabilized and stained with anti-BZLF1 followed by FITC-conjugated anti-mouse IgG_1_. Histograms show the surface MHC-II DR or CD74 expression on latent BZLF1^–^ cells (solid line) and lytic BZLF1^+^ cells (dashed line). The shaded histogram is the isotype control staining. (**C**) The same LCL with a subpopulation of spontaneously lytic cells was first stained for surface MHC-II DR or CD74 as before, then were fixed, permeabilized and stained with anti-VCA followed by FITC-conjugated anti-mouse IgG_1_. Histograms show the surface MHC-II DR or CD74 expression on VCA^–^ cells (solid line) and late lytic VCA^+^ cells (dashed line). The shaded histogram is the isotype control staining.

Similar experiments performed with the Akata-A3 cell line using the DOX inducible vector (Fig 5A) revealed that expression of BZLF1 in these B cells gave similar results to those seen with MJS cells.

To study the surface levels of MHC-II DR and CD74 in the context of lytic cycle in normal EBV-transformed normal B cells, we examined selected LCL cultures that showed a clear subpopulation of cells spontaneously in lytic cycle. These LCLs were stained for surface DR or CD74 on viable cells, then were fixed and permeabilized for staining of intracellular BZLF1. The representative result in Figure 5B shows that the BZLF1^+^ cells spontaneously entering lytic cycle also showed a slight elevation of surface MHC-II DR (around 20%) and a clear reduction of surface CD74 (typically, 30–40%) compared with the BZLF1^–^ latently infected population in the same LCL culture, i.e. mirroring the results obtained with BZLF1-transfected cells in previous experiments. Interestingly, when these same LCL cultures were co-stained for VCA to analyse the minor subpopulation of lytically-infected cells that have progressed to late lytic cycle, both MHC-II DR and CD74 were seen to be downregulated (Figure 5C).

### BZLF1 downregulates CD74 post-translationally

**Figure 6:**
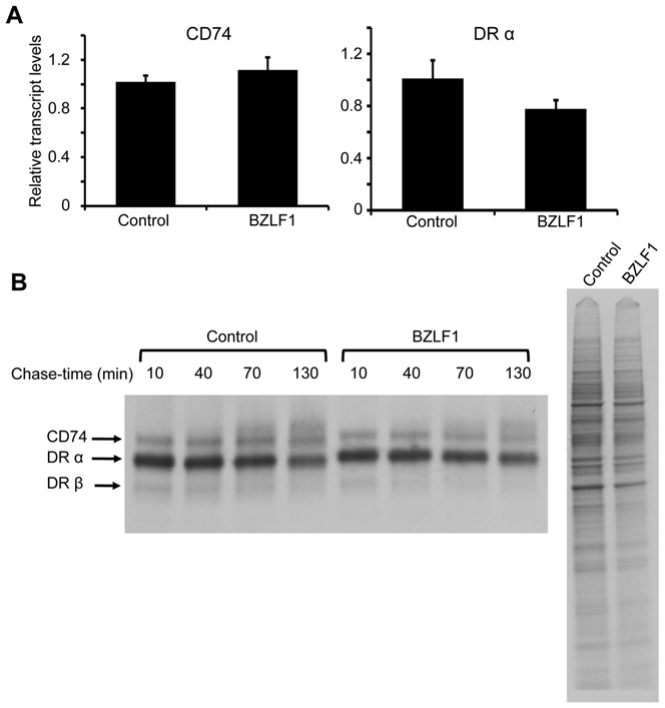
BZLF1 downregulates CD74 post-translationally. (**A**) DRα and CD74 transcripts in total RNA isolated from DOX-induced and anti-NGFR bead-sorted control or BZLF1 transfected Akata-A3 cells, were measured by qRT-PCR assay, which was normalized to measured GAPDH transcripts. (**B**) Dox induced and anti-NGFR bead-sorted control or BZLF1 transfected Akata-A3 cells were metabolically labeled for 15 min with ^35^S-methionine and chased for the indicated time periods. After lysis in NP-40 detergent buffer, samples were immunoprecipitated with mouse anti-CD74. Samples were separated by 12% SDS/PAGE gel, dried and exposed to autoradiography (left panel). Aliquots of the whole cell lysates before chasing were analyzed (right panel) and served as a loading control.

As both MHC-II and CD74 can be transcriptionally regulated by CIITA [22], and BZLF1 was previously reported to transcriptionally repress the CIITA promoter [17], we investigated whether BZLF1 in our experiments might be modulating CD74 transcription via CIITA. Using HEK293 as a model CIITA^–^/MHC-II^–^/CD74^–^ line, we overexpressed CIITA from a heterologous promoter, which in turn induced MHC-II and CD74 protein expression. However, when BZLF1 was co-expressed, surface expression of MHC-II DR was slightly elevated and CD74 was markedly downregulated (Figure S3), exactly as seen in our earlier experiments with MJS and B cells. This suggests that BZLF1 is modulating CD74 expression by a CIITA-independent mechanism. Furthermore, in Akata-A3 B cells, whilst MHC-II DR transcripts were slightly reduced following BZLF1 expression, no reduction in CD74 transcripts was observed (Fig 6A). Pulse-chase metabolic labeling experiments with ^35^S-methionine, revealed that BZLF1 has no effect on CD74 translation or protein maturation (Figure 6B). The exact mechanism of CD74 regulation was not actively pursued further, although experiments performed for other reasons (e.g. Figure S4) indicated that BZLF1 may be regulating trafficking of CD74 to or from the plasma membrane.

### Inhibition of immune CD4^+^ T cell recognition by BZLF1 is retained when toxicity is attenuated by vBcl-2

**Figure 7:**
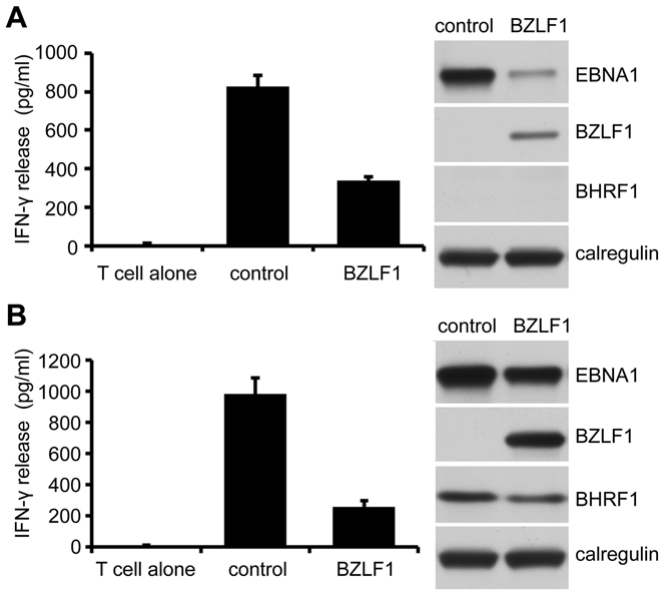
Inhibition of CD4^+^ T cell recognition by BZLF1 is retained when toxicity is reversed by BHRF1. MJS-DRB5*01 cells were co-transfected with a cytoEBNA1 expression vector and IRES-GFP (control) or BZLF1-GFP (BZLF1) expression plasmids, without (**A**) or with (**B**) a BHRF1 expression plasmid. At 24 hr post-transfection, the cells were assayed for recognition by ‘SNP’ CD4^+^ effector T cells (specific for a EBNA1) as in Figure 2A. Error bars for the IFN-γ release in the histograms indicate standard deviation of triplicate cultures. Total cell lysates of the target cell transfections, were analyzed by immunoblotting with antibodies to EBNA1, BZLF1, BHRF1 or calregulin as a loading control.

We next revisited whether BZLF1 retains the ability to impair CD4^+^ T cell recognition when its toxicity is attenuated by BHRF1. MJS cells were co-transfected with cyto-EBNA1 target antigen plasmid and control-IRES-GFP or BZLF1-GFP plasmid vectors without (Figure 7A) or with (Figure 7B) BHRF1 expression plasmid. As in earlier experiments, immune CD4^+^ T cell recognition of the processed EBNA1 target was substantially impaired by expression of BZLF1 in the absence of BHRF1 (Figure 7A, histogram), which correlated with a clear reduction in the amount of EBNA1 antigen expression (Figure 7A, blots). However, when co-expressed with BHRF1 the BZLF1 had no significant effect on the levels of EBNA1 target antigen (Figure 7B, blots), and the immune CD4^+^ T cell recognition was inhibited to an even greater extent than when BHRF1 was not expressed (Figure 7A, histogram).

These data demonstrate that BZLF1 can indeed interfere with MHC-II antigen presentation to cause a substantial impairment of CD4^+^ effector T cell recognition.

### Downregulation of CD74 is a mechanism for impaired CD4^+^ T cell recognition

As BZLF1 impairs T cell recognition without downregulating MHC-II DR expression, we considered it likely that the downregulation of CD74 might be responsible by qualitatively altering the MHC-II/peptide complexes available at the cell surface. To directly test this, we first generated an MJS line in which CD74 was over-expressed from a strong heterologous promoter, to see if we could maintain high levels of surface CD74 after BZLF1 expression and reverse the impaired CD4^+^ T cell recognition. However, despite massive over-expression of total cellular CD74, the amount of CD74 at the cell surface was barely increased; and the ability of BZLF1 to downregulate surface CD74 was unaffected (Figure S4). This is consistent with BZLF1 downregulating surface CD74 by a post-translational trafficking mechanism, but the experiment was otherwise uninformative.

**Figure 8:**
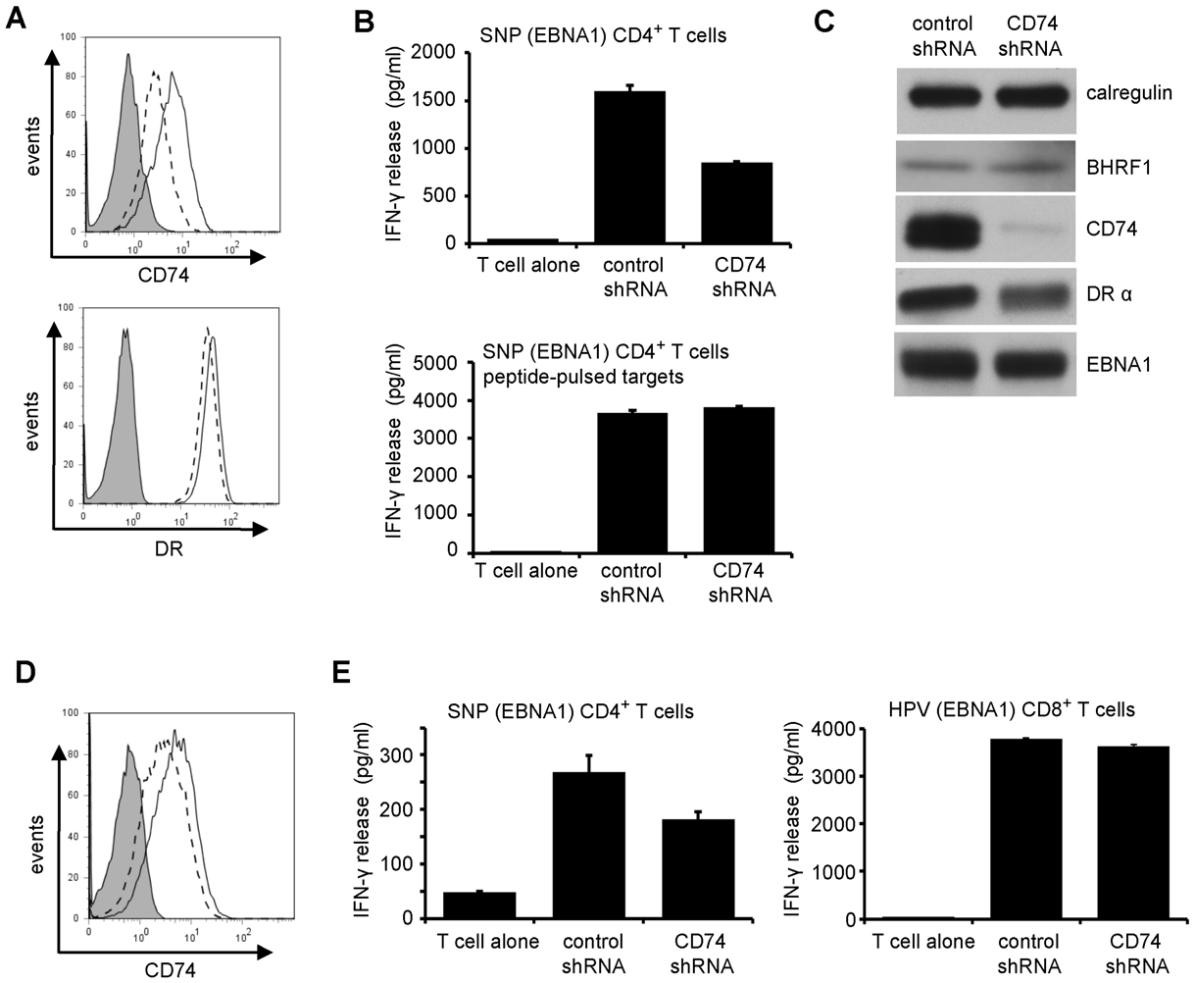
Downregulation of CD74 is sufficient to impair CD4^+^ T cell recognition. (**A**) MJS-BHRF1 cells were transduced with control shRNA or CD74 shRNA lentivirus and selected with puromycin. The stable cell lines were stained with PE-conjugated anti-DR or with PE-conjugated anti-CD74, then analyzed by flow cytometry. Histograms show the surface MHC-II DR or CD74 expression on control shRNA cells (solid line) and CD74 shRNA cells (dashed line). The shaded histogram indicates isotype control staining. (**B**) The control shRNA and CD74 shRNA expressing MJS-BHRF1 cells were co-transfected with a cytoEBNA1 expression vector and DRB5*01 β chain expression plasmids. At 24 h post-transfection, the cells were assayed for recognition by ‘SNP’ CD4^+^ effector T cells specific for a EBNA1 (upper graph). Replicate samples of transfected cells were pulsed with ‘SNP’ peptide to serve as a control (lower graph). Error bars for the IFN-γ release in the histograms indicate standard deviation of triplicate cultures. (**C**) Total cell lysates of the MJS-BHRF1 target cell transfections, were analyzed by immuno-blotting with antibodies to BHRF1, CD74, DRα, EBNA1, or calregulin as a loading control. (**D**) LCLs transformed with B95.8 EBV were transduced with control shRNA or CD74 shRNA lentivirus and selected with puromycin. The stable cell lines were stained with PE-conjugated CD74, then analyzed by flow cytometry. Histograms show the surface CD74 expression on control shRNA cells (solid line) and CD74 shRNA cells (dashed line), with isotype control antibody background staining shaded grey. (**E**) Control shRNA and CD74 shRNA expressing LCLs were assayed for recognition by EBNA1-specific ‘SNP’ CD4^+^ effector T cells (left graph) and ‘HPV’ CD8^+^ effector T cells (right graph).

We next carried out the reverse experiment, asking whether downregulation of CD74 by itself was sufficient to impair CD4^+^ T cell recognition. Again, we first tested this in MJS cells, using an shRNA inhibition approach. Successful knockdown of CD74 in MJS cells was associated with considerable cell death. However, using MJS-BHRF1 cells, we achieved efficient knockdown of CD74 and retained cell viability (Figure 8A and 8C). Importantly, knockdown of CD74 was associated with significant impairment of CD4^+^ T cell recognition of transiently expressed cyto-EBNA1 (Figure 8B, upper histogram). This was a specific effect, as pre-incubation of control and CD74-KO targets with saturating concentrations of synthetic EBNA1 target peptide resulted in both targets being equally-well recognised (Figure 8B, lower histogram). Immunoblots confirmed that the downregulation of CD74 in this experiment had no effect on the expression of the EBNA1 target antigen (Figure 8C). Finally, shRNA-mediated knockdown of CD74 in EBV-transformed LCLs (Figure 8D) resulted in a similarly impaired recognition by CD4^+^ effector T cells (Figure 8E, left histogram). The specificity of this effect in LCLs was demonstrated by the observation that CD74 knockdown had no effect on CD8^+^ effector T cell recognition (Figure 8E, right histogram).

## Discussion

We have demonstrated that the EBV-encoded BZLF1 protein can interfere with recognition by immune CD4^+^ effector T cells, but it comes at a cost of toxicity. During EBV lytic cycle, this toxic property of BZLF1 can be overcome by expression of the vBcl-2 BHRF1 at an early stage of lytic infection. Unexpectedly, the impaired CD4^+^ effector T cell recognition did not correlate with levels of surface MHC-II molecules, but rather with a marked downregulation of CD74 (Ii) on the surface of target cells. CD74 not only facilitates appropriate peptide loading to MHC-II complexes in the endolysosomal vesicles [23]–[25] but, as a surface receptor for Macrophage Migration Inhibitory Factor (MIF), it also enhances cell survival through transcriptional upregulation of Bcl-2 family members [26], [27]. Therefore, downregulation of CD74 is a likely mechanism accounting for both the immune-evasion and death-inducing functions of BZLF1.

CD74 and MHC-II genes can both be regulated by the transcription factor, CIITA [22], and in many instances CD74 and MHC-II genes appear to be co-ordinately expressed. As CIITA expression is negatively regulated by BZLF1 through the suppression of its promoter [17], we might have expected that BZLF1 would downregulate both CD74 and MHC-II. However, from the present work, it is clear that BZLF1 selectively downregulates CD74. Transcription of CD74 is not solely regulated by CIITA [28], which could potentially explain discordant regulation of CD74 and MHC-II genes. Nevertheless, we found that the dominant mechanism by which BZLF1 downregulates CD74 was in fact post-translational.

Although BZLF1 can inhibit CIITA transcription [17], we consistently found cell surface MHC-II DR to be slightly elevated in BZLF1 expressing EBV-negative cells (Figure 4D, 5A). This mirrors what is observed following synchronous induction of lytic cycle in EBV-positive B cells, where cell surface MHC-II DR is initially elevated although it then falls between 12 to 24 h post-induction to a level that is around 40% of that in latently infected cells [29]. In the present study, we also observed that the MHC-II DR is slightly elevated in BZLF1^+^ cells in spontaneously lytic LCLs (Figure 5B), but that the level of surface DR was reduced in the minor subpopulation of cells expressing late viral capsid antigen (Figure 5C). Together, these results suggest that the initial rise in surface MHC-II DR expression in lytically infected cells is likely to be due to BZLF1 expression, while the later reduction in MHC-II DR in lytic cycle may be due to BGLF5, which acts as a host shutoff protein and contributes to immune evasion [2], [3], and/or a delayed effect of BZLF1-mediated inhibition of CIITA.

CD74 is a polypeptide involved in the transport and peptide loading of MHC-II molecules [23]–[25], [30]. Newly synthesized MHC-II α and β chains complex with CD74 (invariant chain) in the endoplasmic reticulum. A cytosolic di-leucine-targeting motif of CD74 directs MHC-II complexes to the endocytic pathway, either directly from the trans-Golgi network or via rapid internalization from the cell surface. The majority of CD74 at the cell surface is physically associated with MHC-II molecules [31] and most, if not all, of immature MHC-II molecules (complex of α chain, β chain and invariant chain) reach the cell surface before entering the peptide-loading compartment [32]. CD74 is rapidly turned over at the cell surface. Downregulation of surface CD74 by BZLF1 may therefore indicate a reduction of available immature MHC-II complexes for processing and uptake of antigenic peptides in the endosomes, and would account for the marked effect of BZLF1 expression on the MHC-II antigen processing pathway. Indeed, when we targeted expression of CD74 through shRNA, the knockdown of CD74 itself was sufficient to inhibit CD4^+^ T cell recognition (Figure 8).

In addition to serving as a chaperone for MHC-II, CD74 has been reported to play an essential role in B cell maturation [33], which involves activation of transcription mediated by p65 member of the NF-κB family [34]. These two functions of CD74 are genetically separable and map to different regions of the protein [35]. More recently CD74 has been identified as a receptor for MIF, and to promote cell survival and proliferation [26]. Binding of MIF to CD74 triggers activation of the p65 member of the NF-κB family, which in turn trans-activates Bcl-2 family genes, thereby providing the cells with increased survival capacity [27]. Furthermore, antibodies that block MIF/CD74 interaction cause growth inhibition and induction of apoptosis in B-cell lines [36]. BZLF1 is known to inhibit NF-κB p65 activity [37] and is toxic for all CD74^+^ cell lines used in our experiments, a phenomenon that correlated with downregulation of the Bcl-2 and Bcl-xl anti-apoptotic proteins (Figure 3C, 4C). It is notable that we observed no toxicity of BZLF1 in the epithelial cell line, HEK-293, which lacks expression of MHC-II and CD74.

The kinetics of the toxicity of BZLF1 in the EBV-negative Akata-A3 B cell line is such that BZLF1-transfected cells survive only 2 days (Figure 3A). This contrasts with what is observed during the normal physiological process of lytic cycle in the EBV-positive parental Akata line, where cell viability is maintained for at least 4 days after expression of BZLF1 [29]. EBV encodes two vBcl-2 homologs, both of which are expressed early following initiation of lytic cycle by EBV. The best characterized vBcl-2 is BHRF1, a potent anti-apoptotic protein that clearly enhances survival of B lymphocytes [21] and whose molecular mechanisms are beginning to be elucidated [38]. In contrast, it is unclear whether the second vBcl-2, BALF1, actually functions to modulate apoptosis [39]. In the present study, we showed that BHRF1 alone is able to moderate BZLF1 toxicity to an extent that is consistent with the enhanced survival period of cells entering lytic cycle.

In this study we have shown for the first time that the MHC-II antigen presentation is impaired during lytic infection of normal B cells (Figure 1). About 80 antigens are expressed in the EBV lytic cycle, representing a large pool of potential target antigens as reflected in the broad repertoire of EBV-specific CD4^+^ T responses identified, including some clones with cytotoxic activity to these EBV antigens [9], [10]. Therefore, impairment of MHC-II antigen presentation is likely to be crucial for the lytic cycle cells to survive long enough to generate infectious virus progeny. In addition to T cell responses to newly-synthesized early and late antigens in lytic cycle, there are also responses to pre-existing MHC-II/peptide complexes from latent antigens expressed at the time of initiation of lytic cycle. In this context, it is interesting to note that recognition of EBNA1, which is expressed during both latent and lytic infection, by specific CD4^+^ T cells is also impaired following expression of BZLF1 and induction of lytic cycle (Figure 1D).

As BZLF1 is the first EBV antigen to be expressed during lytic cycle, a process that can be sustained for several days before cell death occurs [29], its immune-evasion functions may be paramount in EBV's strategy for attenuating anti-viral responses. In this context, the impairment of the MHC-II antigen presentation pathway by BZLF1 adds to other previously reported immune-modulating properties of BZLF1, notably; inhibition the IFN-gamma signaling pathway [40] and TNF-alpha activation [41], [42] by down-regulation of IFN-γ receptor and TNF-R1. However, with regards to modulation of MHC-II antigen presentation it is likely that multiple EBV genes will cooperate to evade immune CD4^+^ T cell responses, as is seen with MHC-I antigen presentation to CD8^+^ T cells [43]. The exonuclease/host shut-off protein, BGLF5, expressed in early lytic cycle may contribute by degrading MHC-II mRNA transcripts [2], [3] and the late BZLF2 glycoprotein, gp42, may contribute by binding to MHC-II molecules and sterically inhibiting recognition by the T cell receptor of immune CD4^+^ T cells [16].

In summary, this work provides a new paradigm for viral immune evasion of MHC-II presented antigen. Targeting CD74 expression is sufficient to substantially impair MHC-II presentation of antigenic peptides even when levels of MHC-II DR molecules are barely affected. However, as CD74 also serves as an important regulator of cell survival, fresh insight is provided as to the role of vBcl-2 during lytic cycle in B cells. It is widely accepted that BHRF1 prolongs cell survival during lytic cycle to allow sufficient time for production and accumulation of new infectious virions. Now, we suggest that BHRF1 also plays a pivotal role in enabling BZLF1 to attenuate recognition by CD4^+^ T cell responses.

## Materials and Methods

### Plasmids and transfection

A derivative of the DOX-dependent expression vector pRTS-1 [44] was kindly provided by Dr J Mautner, Munich; BZLF1 and a reverse BZLF1 sequence as control were introduced into the vector by standard DNA cloning procedures to create vectors pRTS-CD2-BZLF1 and pRTS-CD2-control. The EBV lytic genes BZLF1, BZLF2, BALF4 were also subcloned into the EcoRI/NotI sites of pCDNA3-IRES-nls-GFP vector. All plasmids were verified by restriction digest and sequence analysis. The pCDNA3-cyto-EBNA1 plasmid was described previously [20]. Transient transfection of MJS cells with plasmid DNA was routinely performed using Lipofectamine 2000 (Invitrogen) according to the manufacturer's instructions. Targets for the T cell recognition assay clone were generated by co-transfection of HLA-DRB5*01 MJS cells with a cyto-EBNA1 expression plasmid and IRES-GFP, BZLF2-GFP, BALF4-GFP or BZLF1-GFP expression plasmids.

### Stable transfection and establishment of pBZLF1-tet cell lines

LCLs were established using the reference B95.8-based recombinant lacking the BZLF1 gene (BZLF1KO) [45]. A doxycycline (DOX)-inducible BZLF1 expression vector, pRTS-CD2-BZLF1, or control vector with the reverse BZLF1 sequence (pRTS-CD2-control) were introduced into LCLs or Akata-A3 by electroporation of 10 µg plasmid DNA into 10^7^ cells in OptiMem medium (Invitrogen) at 280 V and 960 µF using a Biorad electroporation apparatus. Transfected cells were cultured in RPMI-1640 supplemented with 10% fetal calf serum (FCS). After 24 h, the transfected cell population was enriched by staining with OX34 antibody to rat CD2, and positively selected by magnetic cell sorting with anti-mouse IgG2a/b Microbeads and LS columns (Miltenyi Biotech) according to the manufacturer's guidelines. Cells were thereafter expanded and maintained in RPMI 1640 medium supplemented with 10% FCS. BZLF1 expression was induced by addition of 200 ng/ml DOX for 24 h, and the induced cells were positively selected by magnetic cell sorting with anti-NRFR Microbeads and LS columns (Miltenyi Biotech). The purity of the sorted cells was checked with Beckman Coulter XL flow cytometer.

### MJS-DRB5*01 and MJS-BHRF1 cells

The MJS (Mel JuSol) melanoma-derived cell line [46] was maintained in RPMI 1640 medium (Gibco BRL) supplemented with 10% FCS. HLA-DRB5*01 expressing MJS cells were generated by transduction with a DRB5*01 retrovirus vector; a HLA-DRB5*01 β chain gene was cloned into retroviral expression plasmid pQCXIN (Clontech) by standard methods. Vesicular stomatitis virus-pseudotyped retrovirus particles were produced in GP2-293 cells co-transfected with the pVSV-G envelope vector. Virus in the culture supernatant at 72 h was concentrated by ultracentrifugation and used to infect 5×10^5^ target cells overnight. Infected cells were selected with G418 (Invitrogen).

BHRF1 retroviral constructs were engineered by cloning the cDNA encoding BHRF1 into the pLZRS retroviral vector. Immediately downstream from the inserted BHRF1 gene lies an IRES sequence and the marker gene, a truncated nerve growth factor (ΔNGFR). Vesicular stomatitis virus-pseudotyped retrovirus particles were produced as above and used to transduce MJS cells. Transduced cells were magnetically sorted using MACS NGFR-specific beads as directed by the manufacturer (Miltenyi Biotech).

### CD74 knockdown by shRNA lentivirus

The lentivirus plasmids containing a sequence of CD74-specific shRNA or a sequence of scrambled shRNA were purchased from Santa Cruz Biotechnology. Vesicular stomatitis virus-pseudotyped lentivirus particles were produced in FT-293 cells co-transfected with the pVSV-G and Gag-Pol expressing vectors. Virus in the culture supernatant at 72 h was concentrated by ultracentrifugation and used to infect 5×10^5^ target cells overnight. Infected cells were selected with puromycin (Sigma).

### Metabolic labelling and immunoprecipitation

Cells were starved by culturing 10^7^ in 15 ml methionine-free RPMI medium supplemented with 10% dialysed FCS for 1 h at 37°C, then labeled for 15 min with 200 µCi of ^35^S protein labeling mix (PerkinElmer) in a final volume of 1 ml. After two washes with chase medium (normal RPMI medium supplemented with 10% FCS), the cells were resuspended at 2×10^6^ cells/ml and chased at 37°C for the times indicated. Samples containing 2×10^6^ cells were lysed in 400 µl of NP-40 buffer (0.5% Nonidet P-40, 5 mM MgCl_2_ and 50 mM Tris-HCl, pH 7.5) with protease inhibitor cocktail (Sigma) at 4°C for 45 min. Nuclei and insoluble debris were removed by centrifugation, and the supernatants were precleared; first with 1.2 µl normal mouse serum and 20 µl Dynabeads Protein A (Invitrogen) for 2 h at 4°C, and then with 20 µl Dynabeads Protein A and 20 µl Dynabeads protein G at 4°C overnight. The precleared lysates were immunoprecipitated for 2 h with 1 µg of mouse anti-CD74 and 20 µl Dynabeads Protein A plus 20 µl Dynabeads protein G, before washing the beads four times with NET buffer (0.5% NP-40, 150 mM NaCl_2_, 5 mM EDTA and 50 mM Tris-HCl, pH 7.5) and eluting by boiling in reducing gel sample buffer for 5 min. Finally, the samples were separated by SDS-PAGE on 12% Bis-Tris NuPage mini-gels with MOPS buffer (Invitrogen). After the gels were fixed and dried, they were exposed to autoradiographic film.

### Antibodies

Goat antibodies to calregulin (sc6467), mouse anti-Bcl-xl, the mouse anti-CD74 and the mouse anti-DRα were purchased from Santa Cruz Biotechnology. Clone 124 mouse antibody to Bcl-2 [47] was a kind gift from the late David Mason. Antibodies to EBV antigens are described elsewhere [48]. For flow cytometry experiments, phycoerythrin (RPE)-conjugated goat anti-mouse IgG antibodies were purchased from AbD Serotec.

### Flow cytometry analysis of cell surface MHC molecules

Cell surface expression of MHC-II DR or CD74 on viable cells was determined by staining with PE-conjugated anti-DR (AbD Serotec) or anti-CD74 primary antibodies followed with RPE-conjugated goat anti-mouse IgG2a (AbD Serotec). Intracellular staining to detect cells expressing nuclear BZLF1 was performed on 10^6^ cells that were fixed using 100 µl of Ebiosciences Intracellular (IC) Fixative for 1 h on ice, followed by permeabilisation through the addition of Triton X-100 to a final concentration 0.2% for a further 30 min incubation on ice. After extensive washing with PBS, the cells were stained with 1 µg/ml of either MAb BZ.1 (anti-BZLF1) or with an IgG_1_ isotype control MAb for 1 hr at 37°C, followed by a 1∶50-dilution of RPE-conjugated goat anti-mouse IgG1 antibody (AbD Serotec) for 1 hr at 37°C. Stained cells were analyzed on Beckman Coulter XL flow cytometer, and the data processed using Flowjo software (Tree Star).

### Western blotting

Total cell lysates were denatured in reducing sample buffer (final concentration: 2% SDS, 72.5 mM Tris-HCl pH 6.8, 10% glycerol, 0.2 M sodium 2-mercaptoethane-sulfonate, 0.002% bromophenol blue), then sonicated and heated to 100°C for 5 min. Solubilized proteins equivalent to 10^5^ cells/20 µl sample were separated by sodium dodecylsulfate polyacrylamide gel electrophoresis (SDS-PAGE) on 4–12% acrylamide gradient Bis-Tris NuPage mini-gels with MOPS running buffer (Invitrogen). Following electroblotting to polyvinylidene difluoride membranes, immunoblotting with specific primary antibodies followed by detection with appropriate alkaline phosphatase-conjugated secondary antibodies and a CDP-Star™ chemiluminescence detection kit (Tropix, Applied Biosystems) was performed as previously described [5].

### T cell function assays

T cells were grown in 10% FCS in RPMI-1640 medium supplemented with 30% supernatant from the IL-2-producing MLA 144 cell line and 50 U/ml recombinant IL-2. The effector CD4^+^ T cell clones ‘LEK’ (specific for amino acids 126–140 of BXLF2 and restricted through DRB5*01) and ‘SNP’ (specific for amino acids 474–493 of EBNA1 and restricted through DRB5*01) were generated as described elsewhere [10], [20]. The effector CD8^+^ T cell clone ‘HPV’ (specific for aa 407–417 of EBNA1 and restricted through MHC-I B35.01) was described elsewhere [49]. The capacity of CD4^+^ and CD8^+^ T cell clones to recognize target LCLs or MJS cells was measured by IFNγ ELISA (Endogen). Briefly, 10^4^ effector T cells were incubated for 18 h at 37°C in V-bottom microtest plate wells with 10^5^ target cells, before assaying the supernatants for IFN-γ release by ELISA (Endogen) in accordance with the manufacturer's recommended protocol.

### QRT-PCR assay

Total RNA was isolated from cultured cell lines using QIAGEN RNeasy kit and treated with DNase I (Turbo DNA-free kit; Ambion). Quantitative reverse-transcription polymerase chain reaction (QRT-PCR) assays for DRA, CD74, Bcl-2 and BCl-xl were performed with TaqMan® Gene Expression Assays (applied biosystem), duplexed with GAPDH assays for normalization.

## Supporting Information

Figure S1 **BZLF1-expressing LCLs can be recognized by EBV lytic antigen specific CD4 T cells.** The pRTS-CD2-BZLF1 and pRTS-CD2-control vector transfected BZLF1KO LCLs were induced by treatment with DOX for 24 h. The induced cultures were assayed for recognition by various CD4+ effector T cell clones specific for different EBV lytic cycle antigens. The CD4+ T cells used in this figure were: ‘VKF’ effectors specific for amino acid residues 11–25 of BZLF1 protein and restricted through DRB3*01; ‘VKL’ effectors specific for amino acid residues 136–150 of BMRF1 protein and restricted through DRB13*01; ‘LDL’ effectors specific for amino acid residues 61–81 of gp350 protein and restricted through DRB1*15. Induced BZLF1KO LCLs and effector T cells were co-cultured for 18 h, and culture supernatants were tested for the release of IFN-γ as a measure of T cell recognition. All results are expressed as IFN-γ release in pg/ml, and error bars indicate standard deviation of triplicate cultures.
(TIF)

Figure S2 **The toxicity of BZLF1 in EBV negative DG75 B cells can be attenuated by BHRF1.** EBV negative DG75 B cells were transfected with pMAX-GFP expression plasmid alone (solid line), together with pCDNA-BZLF1 expression plasmid (dotted line) or together with pCDNA-BZLF1 and pSG5-BHRF1 expression plasmids (dashed line). All transfection plasmid mixes were bulked to a constant amount of DNA with control vector. Cells were harvested at indicated time points after transfection for analysis of GFP expression by flow cytometry. All results are expressed as the percentage of GFP+ cells, and error bars indicate standard deviation of three independent transfections.
(TIF)

Figure S3 **CD74 can be downregulated by BZLF1 in a CIITA-promoter independent manner.** 293-CIITA cells were generated by transduction with a retrovirus vector. Retroviral constructs were engineered by cloning the cDNA encoding CIITA (accession number EAW85172) into the pLZRS retroviral vector. Immediately downstream from this gene was an IRES and the marker gene, truncated nerve growth factor (ΔNGFR). Vesicular stomatitis virus-pseudotyped retrovirus particles were produced in GP2-293 cells co-transfected with the pVSV-G envelope vector. Virus in the culture supernatant at 72 h was concentrated by ultracentrifugation and used to infect 5×105 target cells overnight. Transduced cells were magnetically sorted using MACS NGFR-specific beads as directed by the manufacturer (Miltenyi Biotech). (A) 293 cells transduced with a control NGFR retrovirus or with a CIITA-IRES-NGFR retrovirus were stained with PE-conjugated anti-DR or with CD74 MAb followed by PE conjugated anti-mouse IgG2a antibody, then analyzed by flow cytometry. Solid lines show the surface MHC-II DR or CD74 expression in 293-CIITA cells. The shaded histogram indicates 293-control cells. (B) Cell lysates prepared from 293 control and 293-CIITA cells were analyzed by immunoblotting using antibodies specific for CIITA, DRα chain, CD74 or calregulin as a loading control. (C)(D) 293-CIITA cells transfected with either IRES-GFP or BZLF1-GFP expression plasmids were stained with PE-conjugated anti-DR (C) or with CD74 MAb followed by PE conjugated anti-mouse IgG2a antibody (D), then analyzed by flow cytometry. Histograms show the surface MHC-II DR or CD74 expression on GFP– cells (solid line) and GFP+ cells (dashed line). The shaded histogram indicates isotype control staining.
(TIF)

Figure S4 **Downregulation of CD74 by BZLF1 cannot be reversed when the CD74 is over expressed from a CMV promoter.** MJS cells with CMV promoter-driven CD74 over-expression were generated by transduction with a retrovirus vector. CD74 cDNA was cloned into retroviral expression plasmid pQCXIH (Clontech) by standard methods. Vesicular stomatitis virus-pseudotyped retrovirus particles, including PQCXIH empty vector and PQCXIH-CD74 were produced in GP2-293 cells co-transfected with the pVSV-G envelope vector. Virus in the culture supernatant at 72 h was concentrated by ultracentrifugation and used to infect 5×105 target cells overnight. Infected cells were selected with Hygromycin (Invitrogen). (A) Cell lysates of MJS-PQCXIH and MJS-CD74 cell lines were analyzed by immunoblotting with antibodies to DRα, CD74, or calregulin as a loading control. (B) MJS-PQCXIH and MJS-CD74 cells were stained with PE-conjugated anti-DR or with PE-conjugated anti-CD74, then analyzed by flow cytometry. Histograms show the surface MHC-II DR or CD74 expression on control MJS-PQCXIH cells (solid line) and MJS-CD74 cells (dashed line). The shaded histogram indicates isotype control staining. (C) MJS-PQCXIH and MJS-CD74 cells were cotransfected with BHRF1 and either IRES-GFP or BZLF1-GFP expression plasmids were stained with PE-conjugated anti-DR or with PE-conjugated anti-CD74, then analyzed by flow cytometry. Histograms show the surface MHC-II DR or CD74 expression on GFP+ population from IRES-GFP transfected cells (solid line) and GFP+ population from the BZLF1-GFP transfected cells (dashed line). The shaded histogram indicates isotype control staining.
(TIF)

## References

[1] Rickinson AB, Kieff E, Knipe DM, Howley PM (2007). Epstein-Barr virus.. Fields Virology.

[2] Rowe M, Glaunsinger B, van Leeuwen D, Zuo J, Sweetman D (2007). Host shutoff during productive Epstein-Barr virus infection is mediated by BGLF5 and may contribute to immune evasion.. Proc Natl Acad Sci U S A.

[3] Zuo J, Thomas W, van Leeuwen D, Middeldorp JM, Wiertz EJ (2008). The DNase of gammaherpesviruses impairs recognition by virus-specific CD8+ T cells through an additional host shutoff function.. J Virol.

[4] Hislop AD, Ressing ME, van Leeuwen D, Pudney VA, Horst D (2007). A CD8+ T cell immune evasion protein specific to Epstein-Barr virus and its close relatives in Old World primates.. J Exp Med.

[5] Zuo J, Currin A, Griffin BD, Shannon-Lowe C, Thomas WA (2009). The Epstein-Barr virus G-protein-coupled receptor contributes to immune evasion by targeting MHC class I molecules for degradation.. PLoS Pathog.

[6] Zuo J, Quinn LL, Tamblyn J, Thomas WA, Feederle R (2011). The Epstein-Barr Virus-Encoded BILF1 Protein Modulates Immune Recognition of Endogenously Processed Antigen by Targeting Major Histocompatibility Complex Class I Molecules Trafficking on both the Exocytic and Endocytic Pathways.. J Virol.

[7] Zeidler R, Eissner G, Meissner P, Uebel S, Tampe R (1997). Downregulation of TAP1 in B Lymphocytes by Cellular and Epstein-Barr Virus-Encoded Interleukin-10.. Blood.

[8] Long HM, Haigh TA, Gudgeon NH, Leen AM, Tsang C-W (2005). CD4+ T-Cell Responses to Epstein-Barr Virus (EBV) Latent-Cycle Antigens and the Recognition of EBV-Transformed Lymphoblastoid Cell Lines.. J Virol.

[9] Adhikary D, Behrends U, Moosmann A, Witter K, Bornkamm GW (2006). Control of Epstein-Barr virus infection in vitro by T helper cells specific for virion glycoproteins.. J Exp Med.

[10] Long HM, Leese AM, Chagoury OL, Connerty SR, Quarcoopome J (2011). Cytotoxic CD4+ T Cell Responses to EBV Contrast with CD8 Responses in Breadth of Lytic Cycle Antigen Choice and in Lytic Cycle Recognition.. J Immunol.

[11] Tomazin R, Boname J, Hegde NR, Lewinsohn DM, Altschuler Y (1999). Cytomegalovirus US2 destroys two components of the MHC class II pathway, preventing recognition by CD4+ T cells.. Nat Med.

[12] Hegde NR, Tomazin RA, Wisner TW, Dunn C, Boname JM (2002). Inhibition of HLA-DR Assembly, Transport, and Loading by Human Cytomegalovirus Glycoprotein US3: a Novel Mechanism for Evading Major Histocompatibility Complex Class II Antigen Presentation.. J Virol.

[13] Odeberg J, Plachter B, Branden L, Soderberg-Naucler C (2003). Human cytomegalovirus protein pp65 mediates accumulation of HLA-DR in lysosomes and destruction of the HLA-DR **α**-chain.. Blood.

[14] Temme S, Eis-Hübinger AM, McLellan AD, Koch N (2010). The Herpes Simplex Virus-1 Encoded Glycoprotein B Diverts HLA-DR into the Exosome Pathway.. J Immunol.

[15] Ressing ME, van Leeuwen D, Verreck FAW, Gomez R, Heemskerk B (2003). Interference with T cell receptor-HLA-DR interactions by Epstein-Barr virus gp42 results in reduced T helper cell recognition.. Proc Natl Acad Sci U S A.

[16] Ressing ME, van Leeuwen D, Verreck FAW, Keating S, Gomez R (2005). Epstein-Barr Virus gp42 Is Posttranslationally Modified To Produce Soluble gp42 That Mediates HLA Class II Immune Evasion.. J Virol.

[17] Li D, Qian L, Chen C, Shi M, Yu M (2009). Down-Regulation of MHC Class II Expression through Inhibition of CIITA Transcription by Lytic Transactivator Zta during Epstein-Barr Virus Reactivation.. J Immunol.

[18] Paludan C, Schmid D, Landthaler M, Vockerodt M, Kube D (2005). Endogenous MHC Class II Processing of a Viral Nuclear Antigen After Autophagy.. Science.

[19] Zhou D, Li P, Lin Y, Lott JM, Hislop AD (2005). Lamp-2a Facilitates MHC Class II Presentation of Cytoplasmic Antigens.. Immunity.

[20] Leung CS, Haigh TA, Mackay LK, Rickinson AB, Taylor GS (2010). Nuclear location of an endogenously expressed antigen, EBNA1, restricts access to macroautophagy and the range of CD4 epitope display.. Proc Natl Acad Sci U S A.

[21] Henderson S, Huen D, Rowe M, Dawson C, Johnson G (1993). Epstein-Barr virus-coded BHRF1 protein, a viral homologue of Bcl-2, protects human B cells from programmed cell death.. Proc Natl Acad Sci U S A.

[22] Chang CH, Flavell RA (1995). Class II transactivator regulates the expression of multiple genes involved in antigen presentation.. J Exp Med.

[23] Rocha N, Neefjes J (2008). MHC class II molecules on the move for successful antigen presentation.. EMBO J.

[24] van den Hoorn T, Paul P, Jongsma MLM, Neefjes J (2011). Routes to manipulate MHC class II antigen presentation.. Curr Opin Immunol.

[25] Roche PA, Teletski CL, Stang E, Bakke O, Long EO (1993). Cell surface HLA-DR-invariant chain complexes are targeted to endosomes by rapid internalization.. Proc Natl Acad Sci U S A.

[26] Starlets D, Gore Y, Binsky I, Haran M, Harpaz N (2006). Cell-surface CD74 initiates a signaling cascade leading to cell proliferation and survival.. Blood.

[27] Lantner F, Starlets D, Gore Y, Flaishon L, Yamit-Hezi A (2007). CD74 induces TAp63 expression leading to B-cell survival.. Blood.

[28] Zhu L, Jones PP (1990). Transcriptional control of the invariant chain gene involves promoter and enhancer elements common to and distinct from major histocompatibility complex class II genes.. Mol Cell Biol.

[29] Ressing ME, Keating SE, van Leeuwen D, Koppers-Lalic D, Pappworth IY (2005). Impaired Transporter Associated with Antigen Processing-Dependent Peptide Transport during Productive EBV Infection.. J Immunol.

[30] Romagnoli P, Germain RN (1994). The CLIP region of invariant chain plays a critical role in regulating major histocompatibility complex class II folding, transport, and peptide occupancy.. J Exp Med.

[31] Moldenhauer, Henne, Karhausen, MÖLler (1999). Surface-expressed invariant chain (CD74) is required for internalization of human leucocyte antigen-DR molecules to early endosomal compartments.. Immunology.

[32] Ong, Goldenberg, Hansen, Mattes (1999). Cell surface expression and metabolism of major histocompatibility complex class II invariant chain (CD74) by diverse cell lines.. Immunology.

[33] Shachar I, Flavell RA (1996). Requirement for Invariant Chain in B Cell Maturation and Function.. Science.

[34] Matza D, Wolstein O, Dikstein R, Shachar I (2001). Invariant Chain Induces B Cell Maturation by Activating a TAFII105-NF-kB-dependent Transcription Program.. J Biol Chem.

[35] Matza D, Lantner F, Bogoch Y, Flaishon L, Hershkoviz R (2002). Invariant chain induces B cell maturation in a process that is independent of its chaperonic activity.. Proc Natl Acad Sci U S A.

[36] Stein R, Qu Z, Cardillo TM, Chen S, Rosario A (2004). Antiproliferative activity of a humanized anti-CD74 monoclonal antibody, hLL1, on B-cell malignancies.. Blood.

[37] Morrison TE, Kenney SC (2004). BZLF1, an Epstein-Barr virus immediate-early protein, induces p65 nuclear translocation while inhibiting p65 transcriptional function.. Virology.

[38] Kvansakul M, Wei AH, Fletcher JI, Willis SN, Chen L (2010). Structural Basis for Apoptosis Inhibition by Epstein-Barr Virus BHRF1.. PLoS Pathog.

[39] Marshall WL, Yim C, Gustafson E, Graf T, Sage DR (1999). Epstein-Barr Virus Encodes a Novel Homolog of the bcl-2 Oncogene That Inhibits Apoptosis and Associates with Bax and Bak.. J Virol.

[40] Morrison TE, Mauser A, Wong A, Ting JP, Kenney SC (2001). Inhibition of IFN-gamma signaling by an Epstein-Barr virus immediate-early protein.. Immunity.

[41] Morrison TE, Mauser A, Klingelhutz A, Kenney SC (2004). Epstein-Barr virus immediate-early protein BZLF1 inhibits tumor necrosis factor alpha-induced signaling and apoptosis by downregulating tumor necrosis factor receptor 1.. J Virol.

[42] Bristol JA, Robinson AR, Barlow EA, Kenney SC (2010). The Epstein-Barr virus BZLF1 protein inhibits tumor necrosis factor receptor 1 expression through effects on cellular C/EBP proteins.. J Virol.

[43] Rowe M, Zuo J (2010). Immune responses to Epstein-Barr virus: molecular interactions in the virus evasion of CD8+ T cell immunity.. Microbes Infect.

[44] Kelly GL, Long HM, Stylianou J, Thomas WA, Leese A (2009). An Epstein-Barr Virus Anti-Apoptotic Protein Constitutively Expressed in Transformed Cells and Implicated in Burkitt Lymphomagenesis: The Wp/BHRF1 Link.. PLoS Pathog.

[45] Feederle R, Kost M, Baumann M, Janz A, Drouet E (2000). The Epstein-Barr virus lytic program is controlled by the co-operative functions of two transactivators.. EMBO J.

[46] Johnson JP, Demmer-Dieckmann M, Meo T, Hadam MR, Riethmüller G (1981). Surface antigens of human melanoma cells defined by monoclonal antibodies. I. Biochemical characterization of two antigens found on cell lines and fresh tumors of diverse tissue origin.. Eur J Immunol.

[47] Pezzella F, Tse AG, Cordell JL, Pulford KA, Gatter KC (1990). Expression of the bcl-2 oncogene protein is not specific for the 14;18 chromosomal translocation.. Am J Pathol.

[48] Croft NP, Shannon-Lowe C, Bell AI, Horst Dl, Kremmer E (2009). Stage-Specific Inhibition of MHC Class I Presentation by the Epstein-Barr Virus BNLF2a Protein during Virus Lytic Cycle.. PLoS Pathog.

[49] Blake N, Lee S, Redchenko I, Thomas W, Steven N (1997). Human CD8+ T cell responses to EBV EBNA1: HLA class I presentation of the (Gly-Ala)-containing protein requires exogenous processing.. Immunity.

