# Supplementary figures and images for: Epstein-Barr Virus Evades CD4+ T Cell Responses in Lytic Cycle through BZLF1-mediated Downregulation of CD74 and the Cooperation of vBcl-2
Source: PLoS Pathog. 2011 Dec 22;7(12):e1002455. doi: 10.1371/journal.ppat.1002455 (PMC3245307; doi:10.1371/journal.ppat.1002455)

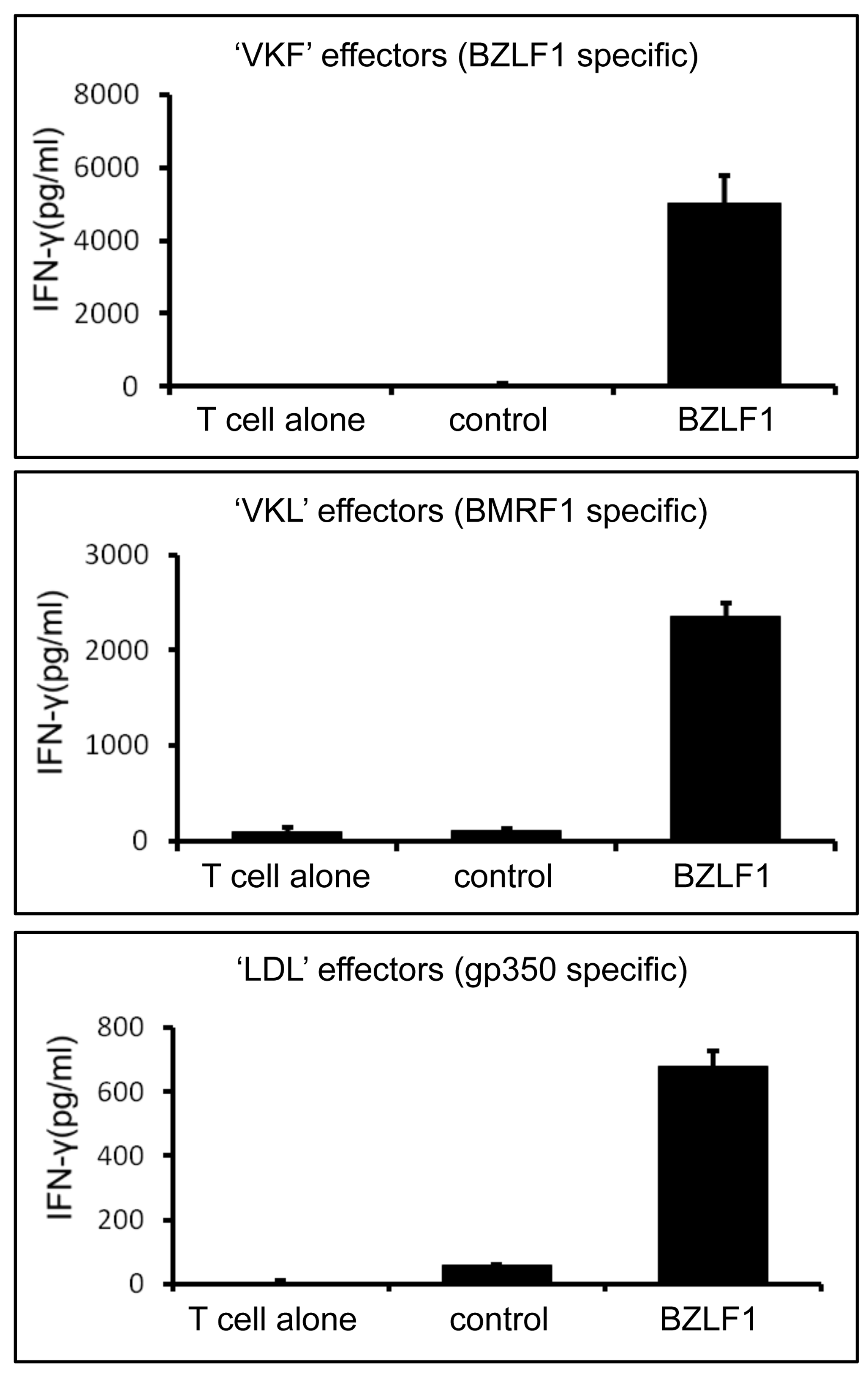

Supplement: Figure S1 — BZLF1-expressing LCLs can be recognized by EBV lytic antigen specific CD4 T cells. The pRTS-CD2-BZLF1 and pRTS-CD2-control vector transfected BZLF1KO LCLs were induced by treatment with DOX for 24 h. The induced cultures were assayed for recognition by various CD4+ effector T cell clones specific for different EBV lytic cycle antigens. The CD4+ T cells used in this figure were: ‘VKF’ effectors specific for amino acid residues 11–25 of BZLF1 protein and restricted through DRB3*01; ‘VKL’ effectors specific for amino acid residues 136–150 of BMRF1 protein and restricted through DRB13*01; ‘LDL’ effectors specific for amino acid residues 61–81 of gp350 protein and restricted through DRB1*15. Induced BZLF1KO LCLs and effector T cells were co-cultured for 18 h, and culture supernatants were tested for the release of IFN-γ as a measure of T cell recognition. All results are expressed as IFN-γ release in pg/ml, and error bars indicate standard deviation of triplicate cultures. (TIF) [file ppat.1002455.s001.tif]

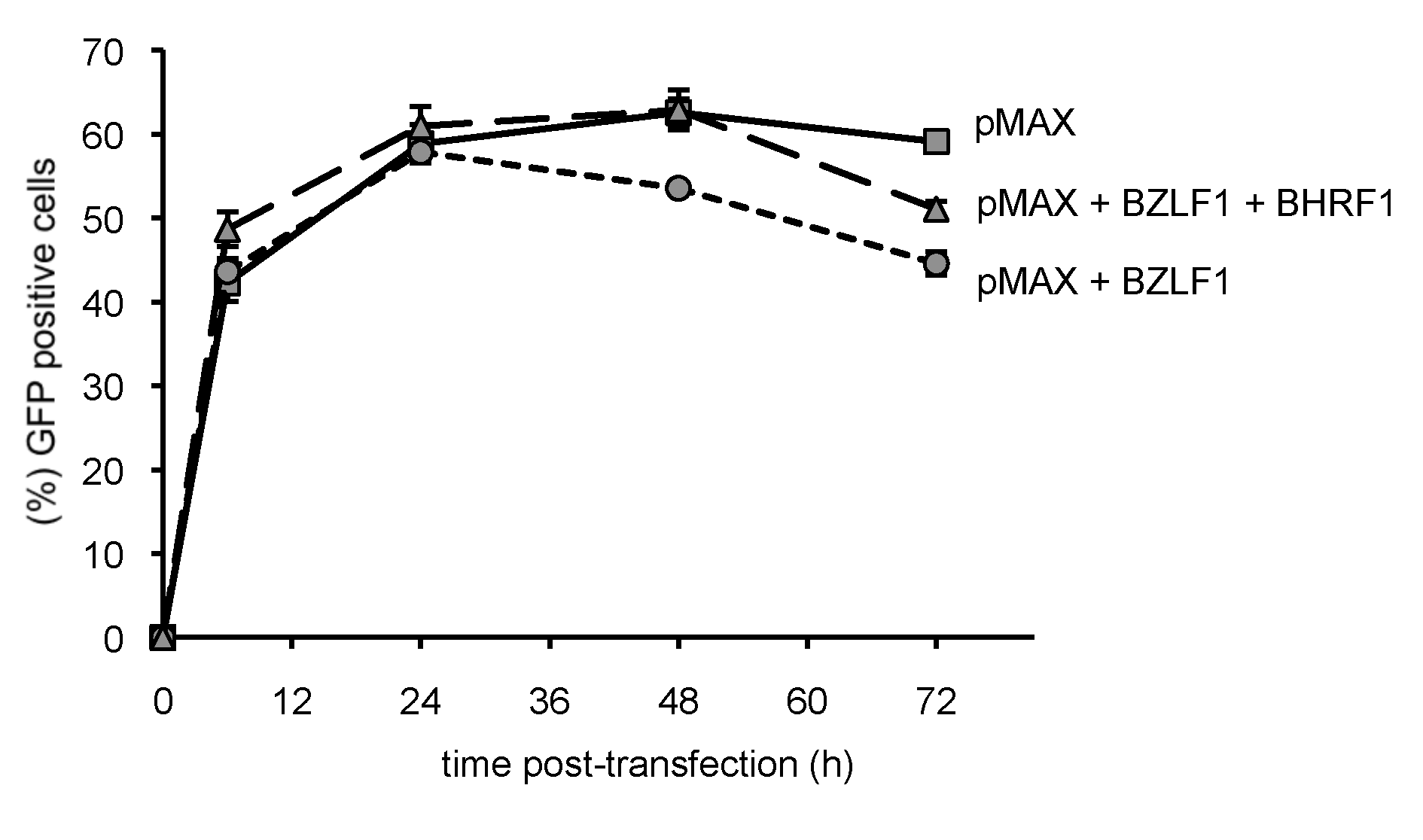

Supplement: Figure S2 — The toxicity of BZLF1 in EBV negative DG75 B cells can be attenuated by BHRF1. EBV negative DG75 B cells were transfected with pMAX-GFP expression plasmid alone (solid line), together with pCDNA-BZLF1 expression plasmid (dotted line) or together with pCDNA-BZLF1 and pSG5-BHRF1 expression plasmids (dashed line). All transfection plasmid mixes were bulked to a constant amount of DNA with control vector. Cells were harvested at indicated time points after transfection for analysis of GFP expression by flow cytometry. All results are expressed as the percentage of GFP+ cells, and error bars indicate standard deviation of three independent transfections. (TIF) [file ppat.1002455.s002.tif]

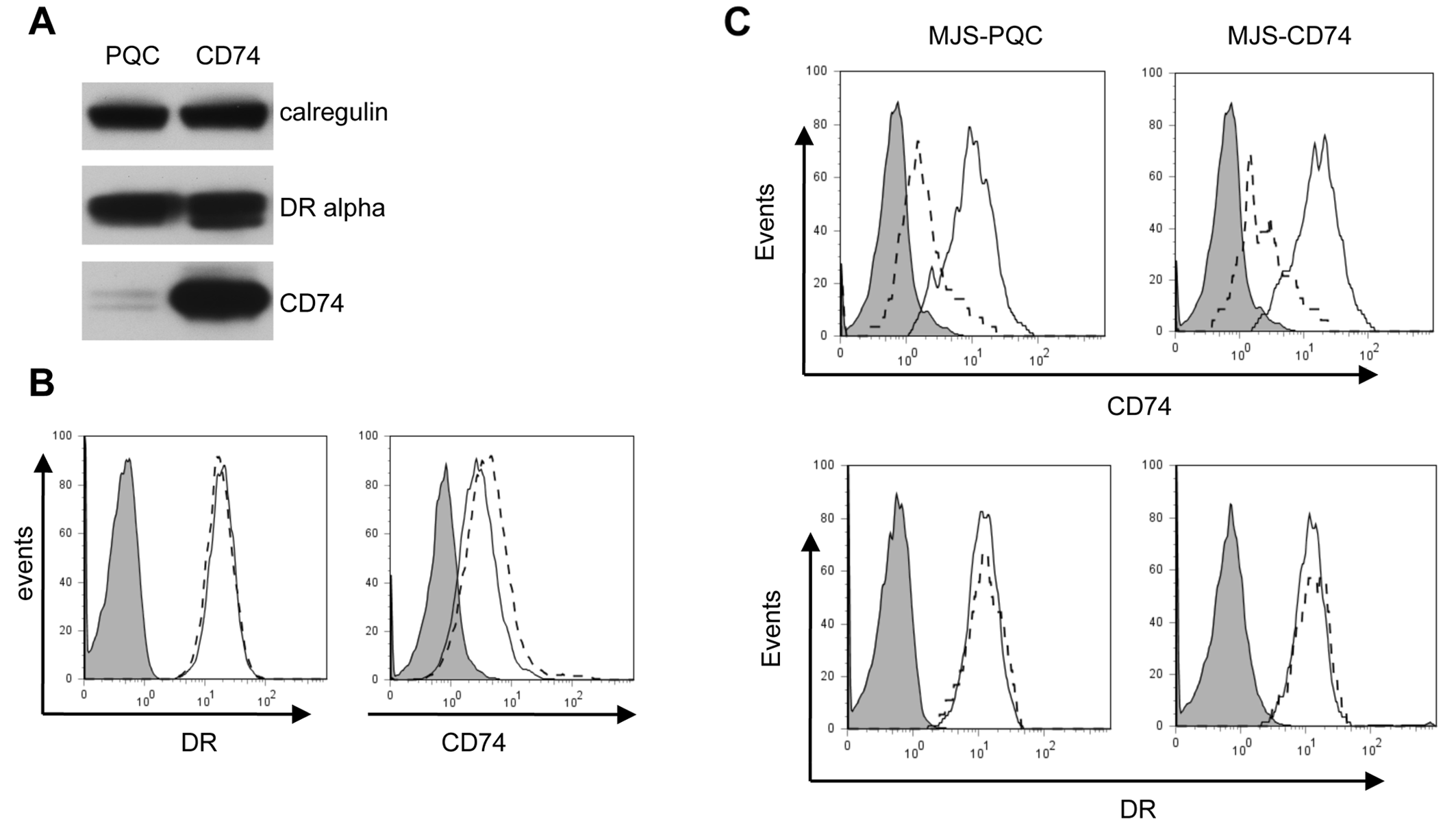

Supplement: Figure S4 — Downregulation of CD74 by BZLF1 cannot be reversed when the CD74 is over expressed from a CMV promoter. MJS cells with CMV promoter-driven CD74 over-expression were generated by transduction with a retrovirus vector. CD74 cDNA was cloned into retroviral expression plasmid pQCXIH (Clontech) by standard methods. Vesicular stomatitis virus-pseudotyped retrovirus particles, including PQCXIH empty vector and PQCXIH-CD74 were produced in GP2-293 cells co-transfected with the pVSV-G envelope vector. Virus in the culture supernatant at 72 h was concentrated by ultracentrifugation and used to infect 5×10^5^ target cells overnight. Infected cells were selected with Hygromycin (Invitrogen). (A) Cell lysates of MJS-PQCXIH and MJS-CD74 cell lines were analyzed by immunoblotting with antibodies to DRα, CD74, or calregulin as a loading control. (B) MJS-PQCXIH and MJS-CD74 cells were stained with PE-conjugated anti-DR or with PE-conjugated anti-CD74, then analyzed by flow cytometry. Histograms show the surface MHC-II DR or CD74 expression on control MJS-PQCXIH cells (solid line) and MJS-CD74 cells (dashed line). The shaded histogram indicates isotype control staining. (C) MJS-PQCXIH and MJS-CD74 cells were cotransfected with BHRF1 and either IRES-GFP or BZLF1-GFP expression plasmids were stained with PE-conjugated anti-DR or with PE-conjugated anti-CD74, then analyzed by flow cytometry. Histograms show the surface MHC-II DR or CD74 expression on GFP+ population from IRES-GFP transfected cells (solid line) and GFP+ population from the BZLF1-GFP transfected cells (dashed line). The shaded histogram indicates isotype control staining. (TIF) [file ppat.1002455.s004.tif]
